# A New and Familiar Treatise on the Structure of the Ear, and on Deafness

**Published:** 1838-07

**Authors:** 


ART. VII.—*A New and Familar Treatise on the Structure of the Ear, and on Deafness.* By A. W. WEBSTER, Inventor of the Otaphone, &c.—*London,* 1836. 8vo. pp. 151.

MR. WEBSTER sets out by expressing some expectation that his little treatise will place our knowledge of the organ of hearing one step in advance of its present position, particularly as regards its successful treatment: a consummation much to be desired. Let us see if his expectations or pretensions be realized.

After some preliminary harangue on the erroneous conceptions which, he says, are prevalent in regard to the structure of the ear, he adds, p. 6, "he has presumed to treat it altogether in a novel, but, he trusts, in no less a correct and scientific manner. He has avoided as much as possible all technical phraseology and minuteness of detail, feeling convinced that they indispose the mind from taking an enlarged view of the subject. He hopes by familiarity of language to improve the science, by enlisting in its support and elucidation all those whom the preceding modes of description have hitherto excluded." This announcement, if anything more than the title-page had been wanting, lets us at once into the secret of the nature of the book, and the conclusion which we thus draw regarding it is justified by all that follows. The book, in fact, belongs to the same class of works which make up the whole amount of English medical literature on the ear, and which we have exposed on more than one occasion in this Review. The treatises in question are intended, not to communicate any information, which, considering the ignorance of all scientific knowledge evinced by their authors, were impossible, but, the gullibility and ignorance of the public in those matters being duly calculated on, to lead to a false belief of their skill.

Each author, of course, has something of his own on which he more particularly founds his pretensions: *Mr. Webster is the inventor of the Otaphone!*

According to him, (p. 131,) "the invention called the Otaphone is constructed" to remedy derangement of hearing depending on the state of the auricle. "The instrument supports the depressed parts of the auricle, and thereby conveying more vibrations to the inner ear, derives its name from οτα (ota) the ear, and φωνη (phone) sound. It is formed of pure silver, doubly gilt, which, taking the exact shape of the back of the ear, when supported in its most capacious form, spreads, as it were, a sail for the collection of sonorous impressions." We give Mr. Webster's description of his instrument, and his derivation of its name in his own words, confessing we cannot see the precise object or use of the thing, or in what it greatly differs from what is known by the name of a Wellington ear; but, we may remark, he informs us, the invention is intended as a substitute for the supporting of the ear with the hand, which people are much in the habit of doing when wishing to catch the words of a speaker in large assemblies; therefore, continues Mr. W. (p. 132,) "the principle on which the otaphones act is so natural as to be almost instinctive." So much so that, he tells us, Dr. M'Diarmid, who attended the last polar expedition, informed him, that the only two *Boothians* who were deaf adopted this practice. From this it would appear the Boothians are as good practical aurists as Mr. Webster himself.

Besides the improvement of the hearing, and the genial warmth which, according to the author, the otaphones promote behind the ear, Mr. W., in the true spirit of a proprietor of a paltry patent invention, enumerates one other advantage which they possess, and what this advantage is, the author must himself be allowed to tell (p. 133): "Sometimes, when the ear is very rigid, as is often the case with men, *the Otaphones cause a small crack in the under surface! This should be hailed as an advantage.* Nature supplying the best medicament to heal the wound, which, when it closes, unites the auricle in a new and better position."

